# Multiple Ischemic Strokes Due to Multisystem Inflammatory Syndrome in Adults (MIS-A)

**DOI:** 10.7759/cureus.22103

**Published:** 2022-02-10

**Authors:** Boby Varkey Maramattom

**Affiliations:** 1 Neurology, Aster Medcity, Kochi, IND

**Keywords:** ttp, stroke, multisystem inflammatory syndrome in adults [mis-a], mis-a, neurological complications

## Abstract

Multisystem inflammatory syndrome in adults (MIS-A) is an extremely rare para-infectious or post-infectious complication of coronavirus disease 2019 (COVID-19) that requires prompt recognition and early treatment to avert severe morbidity and mortality.

A 55-year-old woman presented to us with fever, multiple ischemic strokes, thrombocytopenia, elevated inflammatory markers, and multiorgan dysfunction a few days after COVID-19 illness. She was severe acute respiratory syndrome coronavirus 2 (SARS-CoV-2)-negative at admission. MRI showed multiple posterior circulation infarctions. She required intensive treatment with intravenous methylprednisolone (IVMP), intravenous immunoglobulin (IVIg), sustained low-efficiency dialysis (SLED), and plasmapheresis for disease remission.

Initially, her presentation raised concern for thrombotic thrombocytopenic purpura, however, many features raised the suspicion of a multisystem inflammatory syndrome in adults (MIS-A). Our patient had increased levels of D-dimer, fibrinogen, interleukin 6 (IL-6), and large artery thromboembolism, A positive direct Coomb's test was also more suggestive of immune-mediated hemolysis rather than traction hemolysis, which is the pathophysiology of hemolytic anemia in TTP. Furthermore, MIS-A is known to present with gastrointestinal (GI) symptoms, whereas our case reports predominantly neurological symptoms with relative GI sparing. The overall inflammatory milieu secondary to MIS-A would have contributed to the formation of immune thrombosis, which would have embolized up the vertebrobasilar tree. The MR angiogram did not show any atherosclerotic changes, ruling out an atherosclerotic etiology, which is quite common in posterior circulation infarctions. Multiple courses of immunomodulatory treatment and prolonged treatment with steroids led to disease stabilization.

## Introduction

The combination of stroke or neurological dysfunction, fever, acute kidney injury (AKI), and/or multiorgan dysfunction raises the specter of thrombotic thrombocytopenic purpura (TTP) or other thrombotic microangiopathies (TMA). However, in the aftermath of the coronavirus disease 2019 (COVID-19) pandemic, MIS-A should be considered in the differential diagnosis.

Multisystem inflammatory syndrome (MIS) is a recently described para or post-infectious sequala of COVID-19. Initially, it was described in children (MIS-C). Subsequently, it was identified in adults (MIS in adults) or MIS-A. Different case definitions for the diagnosis of MIS-A have been published [1-2].

## Case presentation

A 55-year-old woman was diagnosed with mild COVID-19 illness. On day 16 during convalescence at home, she was found unresponsive in bed. A repeat SARS-CoV-2 reverse transcription-polymerase chain reaction (RT-PCR) was negative.

Magnetic resonance imaging (MRI) showed multiple posterior circulation infarctions. Her inflammatory markers were elevated at admission: lactate dehydrogenase (LDH) 13,168 (135 - 214 U/L), c-reactive protein (CRP) 58 (< 5 mg/L), D-dimer 1200 (< 500 ng/ml), ferritin 3369 (20-250 ng/ml), interleukin-6 (IL-6) -107.7 (0-7 picogram/ml), and fibrinogen 530 (175-400 mg/dl). Platelet counts were reduced at 125 (150-450 K/microliter) and there was evidence of AKI (creatinine 3.3 (0.6-1.1 mg/dl)) and melena. She was started on dual antiplatelets (asprin and clopidogrel). The echocardiogram and Holter monitoring were normal. On day 20, she was transferred to our institute. On examination, she was drowsy, febrile, tachycardic, and had oral mucositis, dense right hemiplegia, and bilateral extensor plantar responses. She was intubated and ventilated and started on enoxaparin, aspirin, and levetiracetam. Chest radiography was within normal limits.

**Figure 1:**
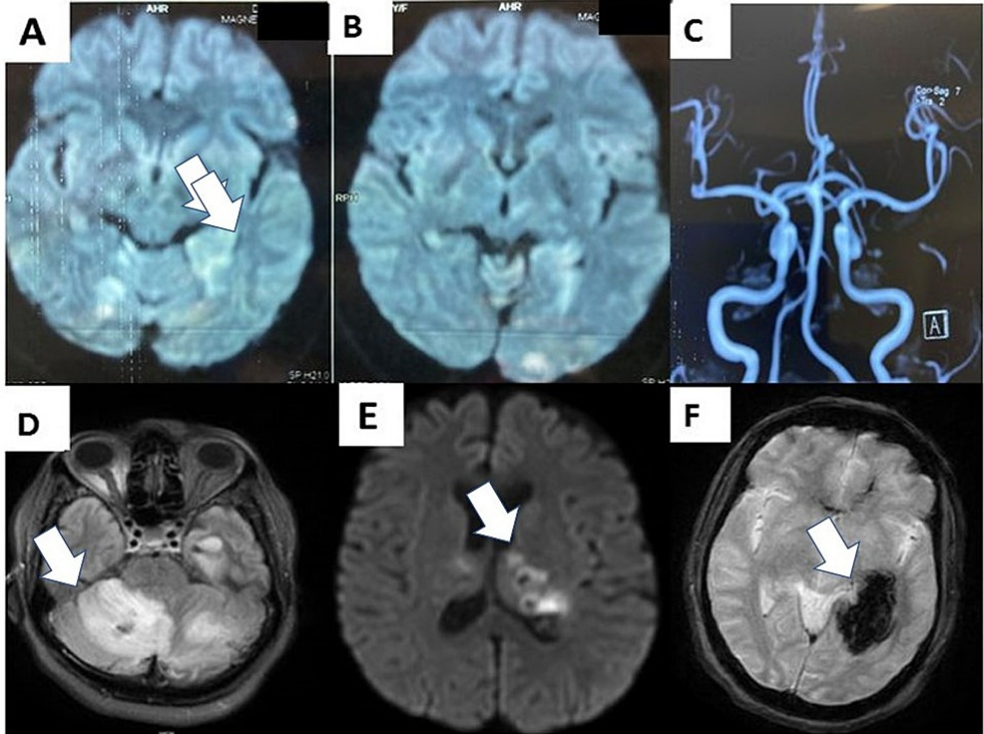
MRI images Panels A and B: Scattered infarcts in the superior cerebellum and bilateral PCA territory on day 16. Panel C: MR angiogram (MRA) with patent intracranial circulation. Panels D and E: Axial MRI diffusion-weighted images showing edema around the cerebellar and thalamic infarcts on day 20. Panel F: Gradient echo MRI images showing a left medial occipital intracerebral hemorrhage.

Repeat MRI on day 20 showed a new left medial occipital intracerebral hemorrhage (Figure 1).

Peripheral smears showed thrombocytopenia without evidence of schistocytes. An ADAMTS-13 level could not be performed due to logistical reasons. The combination of fever, thrombocytopenia, AKI, and neurological involvement was suggestive of thrombotic thrombocytopenic purpura (TTP). Multisystem inflammatory syndrome in adults (MIS-A) was also considered (Tables 1-2).

**Table 1:** Differential diagnosis of MIS-A MIS-A: multisystem inflammatory syndrome in adults

Differential diagnosis of MIS-A
Microangiopathic hemolytic anemia (MAHA)
Severe COVID-19
Staphylococcal toxic shock syndrome
Systemic vasculitis
Bacterial/viral/fungal sepsis
Systemic inflammatory response syndrome (SIRS)
Hemophagocytic lympho-histiocytosis (HLH)/macrophage activation syndrome (MAS)
Infective endocarditis
Rocky Mountain spotted fever
Meningococcaemia
Heparin-induced thrombocytopenia
DRESS syndrome (Drug Rash with Eosinophilia and Systemic Symptoms)
Catastrophic antiphospholipid antibody syndrome (APS)
Adult-onset Still’s disease (AOSD)
Systemic lupus erythematosus (SLE)
Vaccine-induced prothrombotic thrombocytopenic purpura (VIPIT)
Adeno or enteroviral infection

Pan cultures (blood, urine, Bronchoalveolar lavage (BAL)), upper GI endoscopy, and colonoscopy were normal. IV methylprednisolone (IVMP) 1 gm/day was administered for five days (from day 21). Over the next few days, her platelet counts dropped to a nadir of 30,000/microliters. Repeated peripheral smears showed 1-2% schistocytes. She also developed anasarca, hypoalbuminemia, hypernatremia, and hypotension for which a secondary systemic capillary leak syndrome (SCLS) was suspected. After two cycles of sustained low-efficiency dialysis (SLED) and two cycles of plasmapheresis, her hypernatremia and anasarca improved. Anemia, leukopenia, and thrombocytopenia continued to worsen. Bone marrow biopsy showed trilineage hematopoiesis and mild myeloid predominance. A direct Coombs test was positive. Antinuclear antibodies (ANA), antineutrophil cytoplasmic antibody (ANCA), and antiphospholipid antibodies were negative. Myeloproliferative neoplasm (MPN) reflex panel for the JAK2 and CALR genes was negative. On day 26, BAL cultures grew Acinetobacter and Klebsiella species and blood cultures grew Enterobacter cloacae complex, and appropriate antibiotics were started. Intravenous immunoglobulin (IVIg) 2 gm/Kg was administered over five days (day 28).

**Table 2:** CDC criteria for the diagnosis of MIS-A MIS-A: multisystem inflammatory syndrome in adults; CDC: Centers for Disease Control and Prevention; CRP: c-reactive protein

CDC criteria for the diagnosis of MIS-A. The guidelines for the diagnosis of MIS-A include the following five criteria.
1) It should occur in the context of a severe illness requiring hospitalization in an adult ≥21 years.
2) A current or previous SARS-CoV-2 infection should be documented during admission or in the preceding 12 weeks with a positive RT-PCR, antigen, or SARS-CoV-2 antibody test.
3) There should be severe dysfunction of 1 or more extrapulmonary organ systems (e.g., hypotension or shock, cardiac dysfunction, arterial or venous thrombosis or thromboembolism, or acute liver injury).
4) It should be accompanied by laboratory evidence of severe inflammation (e.g., elevated CRP, ferritin, D-dimer, or interleukin-6).
5) There should not be a severe respiratory illness or hypoxia that could explain the above findings. Mild respiratory illness is not against the diagnosis.

On day 35, her fever remitted and platelet counts stabilized. She had residual global aphasia and right hemiparesis. D-dimer and ferritin remained high, and she was continued on IVMP 125 mg/day and low molecular weight heparin. On day 50, apixaban was started and she was transferred for rehabilitation. Her modified Rankin score (mRS) at day 90 was 2.

## Discussion

Severe COVID-19-associated hyperinflammatory syndrome (COVID-HIS) is associated with severe pulmonary involvement resembling acute respiratory distress syndrome (ARDS) and multiorgan dysfunction (MODS). MIS-A has prominent extra-pulmonary involvement with minimal pulmonary findings. The proposed pathophysiologic mechanisms of MIS-A include endothelial damage, thrombo-inflammation, dysregulated host immune responses, dysregulation of the renin-angiotensin-aldosterone system (RAS), or viral persistence in extra-pulmonary tissues [3].

TTP and thrombotic microangiopathy (TMA) have been described in COVID-19 and contribute to COVID-HIS and hypercoagulability. Endothelial damage in COVID-19 leads to highly increased concentrations of Von Willebrand Factor (VWF) and a relative deficiency of ADAMTS13. This relative decrease in the ADAMTS13/VWF ratio exceeds the ADAMTS13 processing capacity leads to excessive exocytosis of ultra-large von Willebrand factor multimers (ULVWF) from endothelial cells and a clinical picture resembling TTP [4]. Hence, plasmapheresis can be considered if there is a high clinical suspicion of TTP or the ADAMTS13 /VWF ratio is < 10.

Our patient had increased D-dimer, fibrinogen, and IL-6 levels and larger artery thromboembolism, high IL-6 levels, and a positive direct Coomb's test (DCT). A positive Coomb's test is more suggestive of immune-mediated hemolysis rather than traction hemolysis, which is seen commonly in TTP [5]. Furthermore, MIS-A is known to present with GI symptoms, whereas our case reports predominantly neurological symptoms with relative GI sparing. The overall inflammatory milieu secondary to MIS-A would have contributed to the formation of an immune thrombosis, which would have embolized up the vertebrobasilar tree. The MR angiogram did not show any atherosclerotic changes, ruling out an atherosclerotic etiology, which is quite common in posterior circulation infarctions.

The treatment of MIS-A is based on small case series and includes aspirin, low molecular weight heparin, inotropes or vasopressors, mechanical ventilation, and mechanical circulatory support (extracorporeal membrane oxygenation). Anti-inflammatory therapies, such as IVIg 2 gm/kg given over one to two days, steroids (IVMP or dexamethasone) are often used. Interleukin 6 (IL-6) inhibitors (anakinra, tocilizumab) are useful if MIS-A is refractory to IVIg and steroids or if there are contraindications to steroids or a macrophage activation syndrome supervenes. Low-moderate dose steroids (1-2 mg/kg/day) may be considered if patients remain persistently febrile and symptomatic even after IVIG or IL-6 inhibitors. Although many patients improve dramatically with short courses of steroids, some patients may require a >2-3-week course of immunomodulatory medications [6]. MIS-A should be considered early, as prompt immune-modulatory treatment is needed in addition to antiplatelets or anticoagulants.

## Conclusions

MIS-A can be associated with severe neurological manifestations. It is important to consider MIS-A when patients present with multiple strokes and multiorgan dysfunction. MIS-A requires intensive treatment with multiple immunomodulators and steroids. It also has a tendency to flare up when immunomodulation is de-escalated. Hence, medium-term, high-dose immunosuppression might be required.
